# Heart-lung transplantation for idiopathic pulmonary arterial hypertension and giant pulmonary artery aneurysm – case report

**DOI:** 10.1186/s13019-020-01221-z

**Published:** 2020-07-13

**Authors:** T. Eadington, K. Santhanakrishnan, R. Venkateswaran

**Affiliations:** 1 Department of Heart and Lung Transplantation, Wythenshawe Hospital, Manchester University NHS Foundation Trust, Manchester, UK; 2 Honorary Senior Lecturer, Division of Cardiovascular Science, University of Manchester, Manchester, UK

**Keywords:** Pulmonary artery aneurysm, Pulmonary hypertension, Heart-lung transplantation, Cardiothoracic transplantation

## Abstract

**Backgound:**

Idiopathic pulmonary arterial hypertension (IPAH) is a rare condition that requires lung transplantation in patients’ refractory to medical therapy. Pulmonary artery aneurysm (PAA) is a documented complication of IPAH however, optimal management and timing of intervention for this rare entity is not well understood.

**Case report:**

We report a case of a 51-year-old female who underwent heart-lung transplantation for IPAH and giant PAA. The extreme size of the PAA and underlying pathology encountered in this case precluded both lung transplantation and conventional aneurysm repair.

**Conclusion:**

This case demonstrates that heart-lung transplantation is a good surgical option for IPAH complicated by giant sized PAA and right heart failure.

## Background

Pulmonary arterial hypertension (PAH) is a chronic lung disorder characterised by a progressive and pathological remodelling of the resistance pulmonary arteries. The resulting pulmonary vasoconstriction causes an abnormal increase in pulmonary vascular resistance, impairing pulmonary circulation and increasing the workload on the heart due to a chronically increased right ventricular afterload [1, 2]. There are several different causes of PAH as described under group 1 of the WHO classification of pulmonary hypertension [3], with the idiopathic subtype seen in this case accounting for approximately 39% of these [4]. Whilst the incidence of the condition is not fully understood, it is rare, with an estimate between 2.5–7.1 cases/million with a female:male predominance of between 1.8–3.6: 1 [5, 6]. Historic studies in the US reported the mean age of onset of IPAH as 36 +/− 15 years [7], however results from more recent registries have reported a much higher mean age of onset (53 +/− 14 years) [8]. PAH has previously been defined by a mean pulmonary arterial pressure at rest of ≥25 mmHg at rest with a pulmonary capillary wedge pressure ≤ 15 mmHg [9]. However, this arbitrary definition remains a point of debate [10].

Pulmonary artery aneurysm (PAA) has been described as a dilatation of the pulmonary artery beyond the normal upper limit of diameter of 29 mm. Some have proposed a definition of dilatation above 4 cm however an exact definition remains unclear [11, 12]. They are a very rare finding with an approximate incidence of 1:14000 [13] and as a result there is currently no agreement on how or when to treat these entities for optimum outcomes. PAA have a wide range of causes both congenital and acquired; however, they do have a strong association with PAH. Aneurysm formation and progression is associated with vessel wall stress and rupture carries an extremely high mortality rate (50–100%) [14].

Further classification of ‘giant’ PAA has been attempted. Some have suggested a cut off-of 6 cm to define the label of ‘giant’ and others 8cm [15, 16]. Optimal management of these very large PAAs is even more difficult to determine. In some cases correction of pulmonary valve distortion has been sufficient to preserve right ventricular function [15] whilst others advocate surgical repair of the PAA when size exceeds 8cm [16]. Surgical repair of large PAA has been described in the presence of pulmonary hypertension [17] however, this is the first report of a patient with IPAH and a PAA of such extreme size with concurrent right heart failure.

## Case presentation

This is the case of a 51-year-old female with a past medical history of IPAH. This had been diagnosed by right heart catheterization 16 years previously when the patient collapsed at home shortly after childbirth. The patient’s condition was stable for many years, being medically managed with intravenous iloprost at 1300 μg once daily. This allowed the patient an acceptable quality of life maintaining a WHO functional class II.

A computed tomography pulmonary angiogram in 2013 diagnosed a 5.5 cm PAA, worst affecting the main and left pulmonary arteries. Right heart catheter performed at this time demonstrated pulmonary artery pressure of 90/39 mmHg (mean 31 mmHg), right ventricular pressure of 93/10, end diastolic diastolic pressure of 20 mmHg and a mean right atrial pressure of 13 mmHg. Due to the progression of the underlying pathology additional pulmonary antihypertensives were added to the treatment plan (sildenafil and ambrisentan). The patient was considered for bilateral orthotopic lung transplantation but after assessment was deemed too clinically well to list for transplant at that time and the patient continued to be monitored by their local hospital.

Clinical deterioration prompted review at our centre in 2019, which identified worsening symptoms of shortness of breath, causing severe limitation of daily function (WHO functional class III-IV), and left shoulder pain. Echocardiography revealed marked progression of the PAA causing dilatation of the pulmonary valve annulus and subsequent severe pulmonary regurgitation. There was also severe right ventricular impairment, moderate tricuspid regurgitation and a 4 cm pericardial effusion lateral to the left ventricular wall. Pulmonary artery pressure was estimated at > 65 mmHg.

**Fig. 1:**
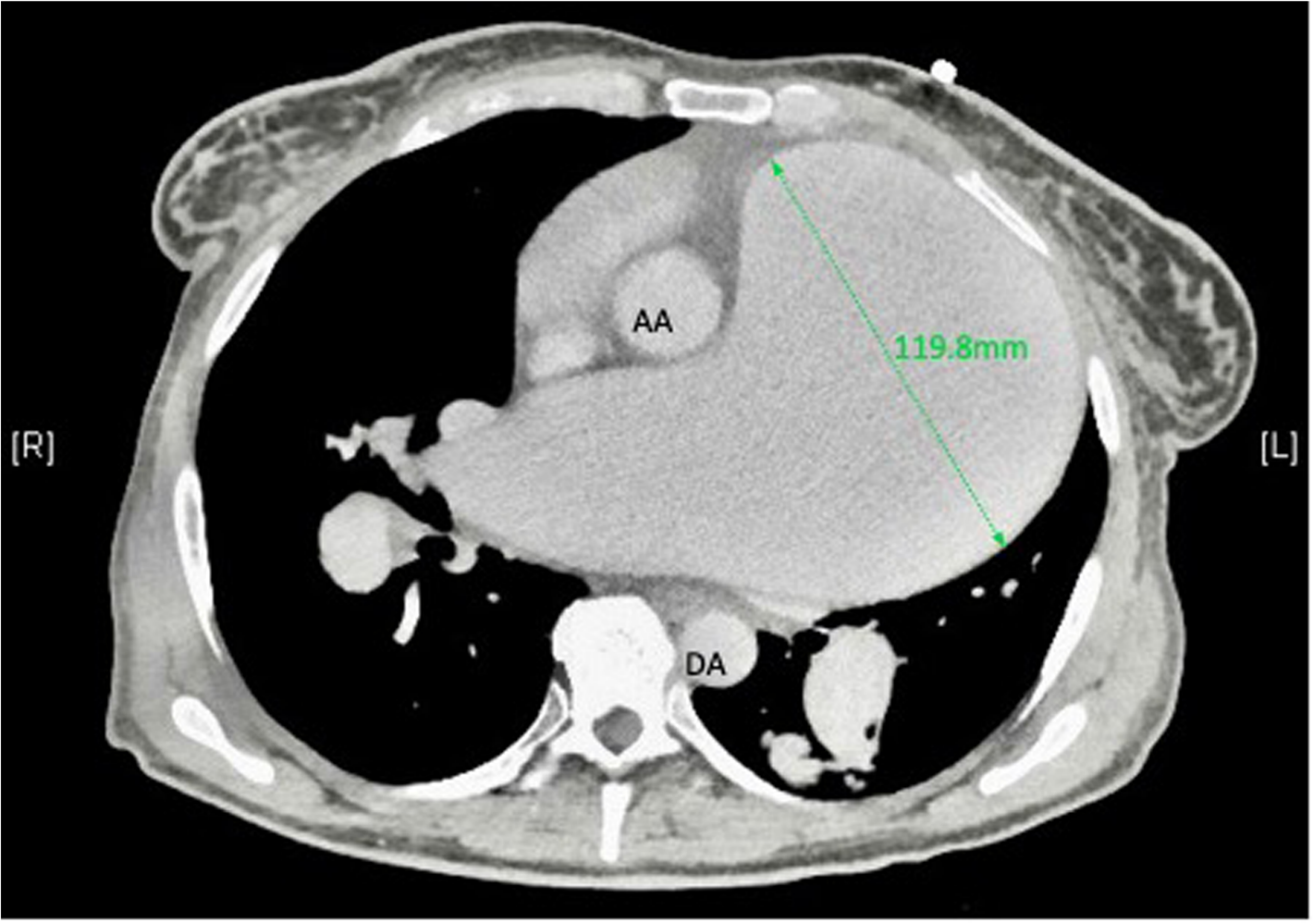
CTPA demonstrating giant pulmonary artery aneurysm (green arrow). AA = ascending aorta, DA = descending aorta

**Fig. 2:**
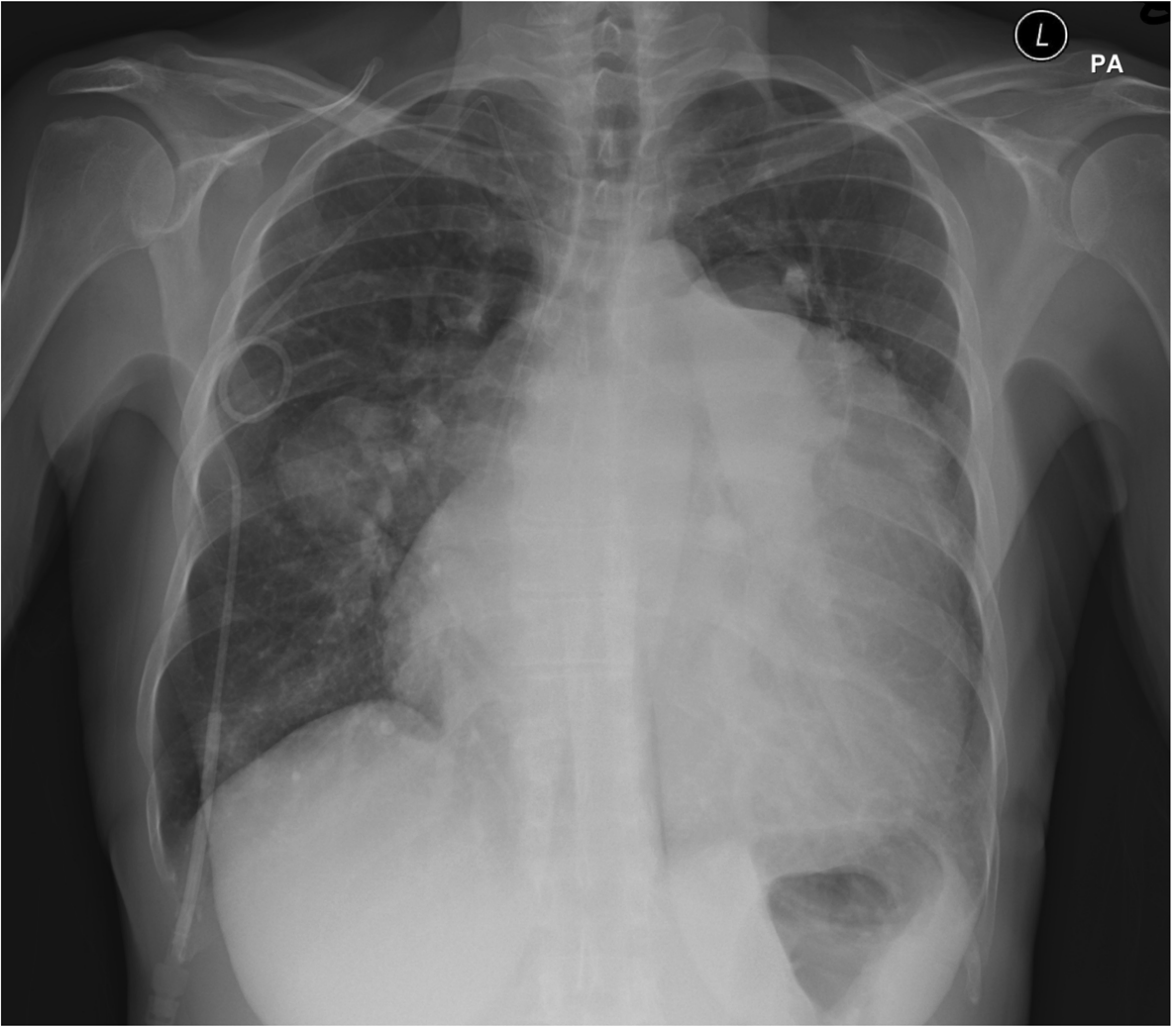
Pre-operative chest xray demonstrating cardiomegaly, marked mediastinal enlargement occupying the left thorax and deviation of the carina.

Computed tomography pulmonary angiography confirmed a 12 cm aneurysm of the pulmonary artery mainly affecting the main and left pulmonary arteries. There was also a 6 cm aneurysm of the right pulmonary artery (Fig. 1). Cardiac magnetic resonance imaging confirmed a right ventricular ejection fraction of 23% (this had been 40% at the previous assessment). A chest x-ray demonstrated marked cardiomegaly, tracheal deviation, and an extremely widened mediastinum extending into the left hemi-thorax (Fig. 2).

Due to the presence of a giant PAA, severe pulmonary regurgitation and a 4 cm pericardial effusion, it was decided to list the patient for heart-lung transplantation. Episodes of right heart failure were managed with inotropes and the patient was maintained on pulmonary antihypertensive medications and diuretics whilst awaiting transplantation.

**Fig. 3:**
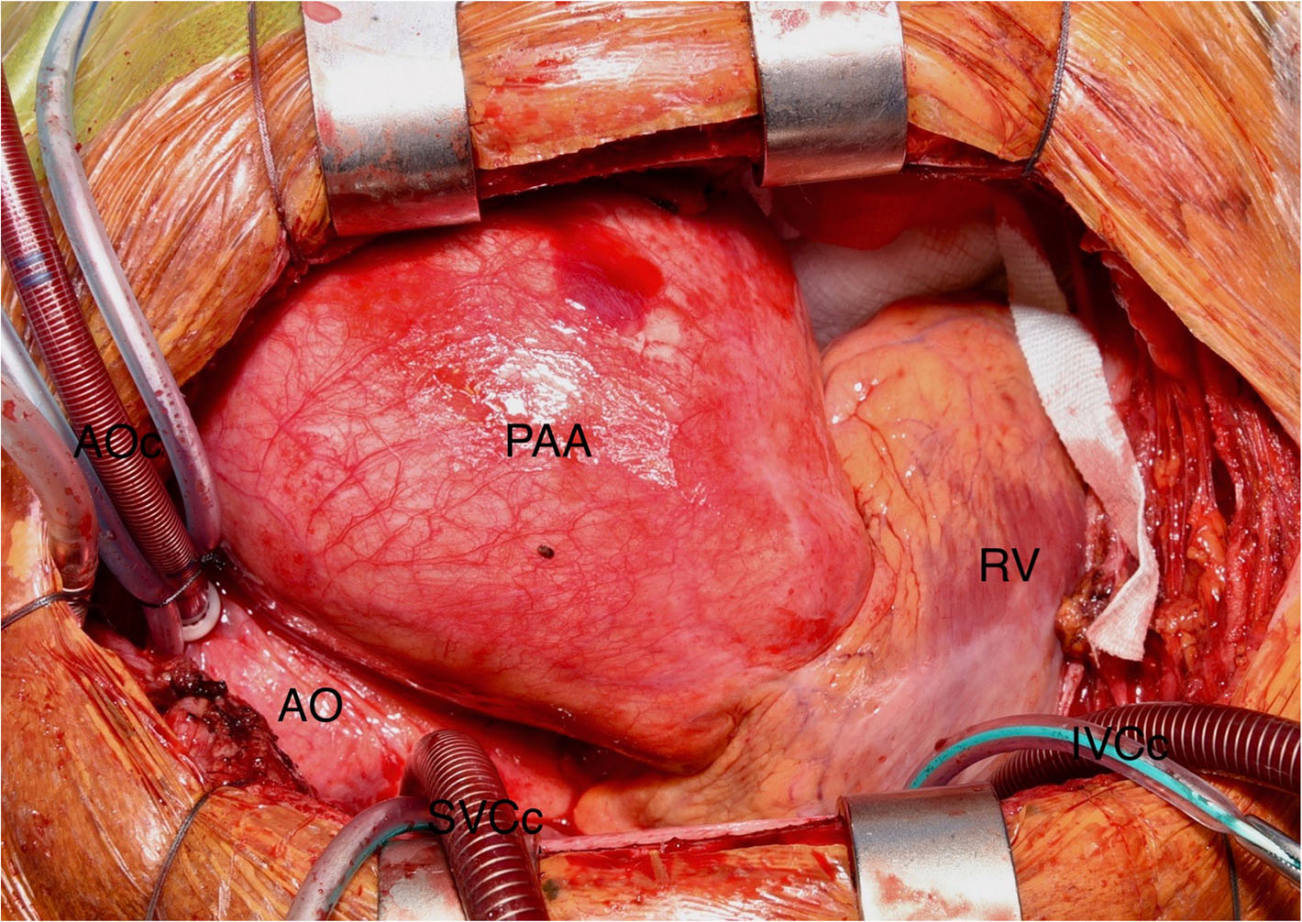
Intra-operative image of giant pulmonary artery aneurysm (PAA). Note the lateral displacement of the aorta (AO) and the inferior displacement of the heart (RV). AOc = aortic cannula, SVCc = superior vena cava cannula, IVCc = inferior vena cava cannula

**Fig. 4:**
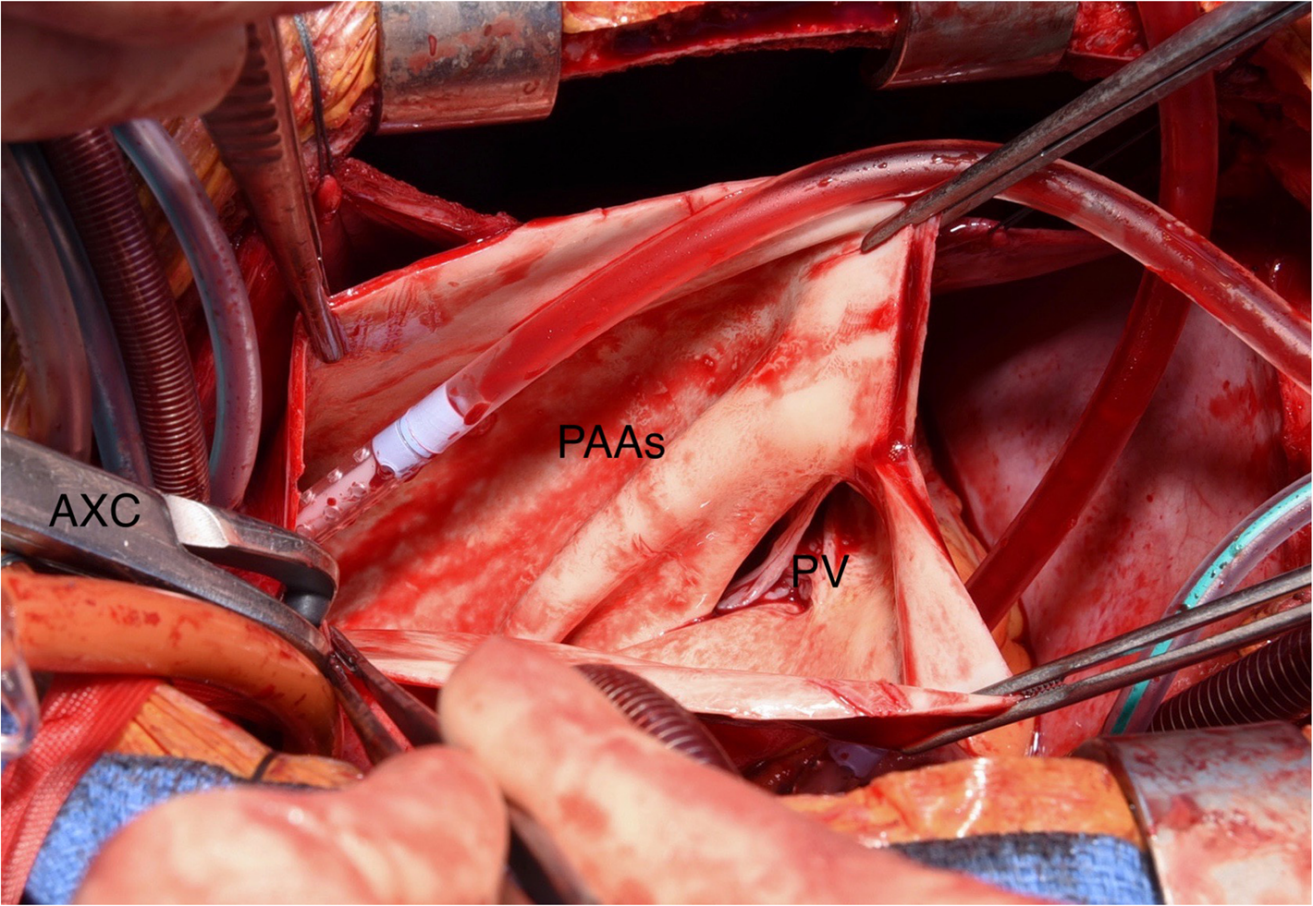
Intra-operative image showing dissection of the aneurysm sac (PAAs) during explantation of the recipient heart. Note the distorted pulmonary valve (PV). AXC = aortic cross-clamp

**Fig. 5:**
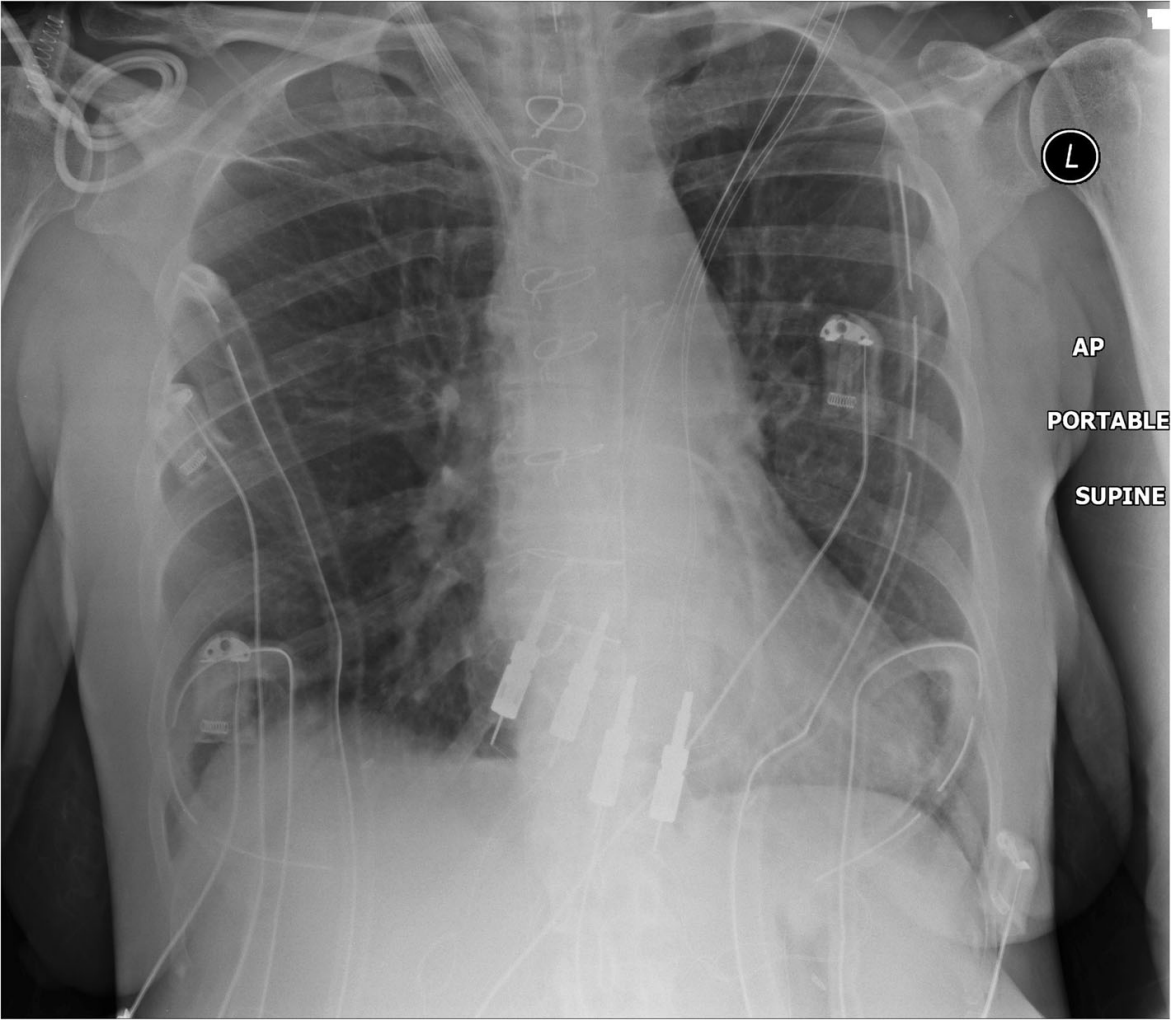
Post-operative chest x-ray showing the new heart-lung bloc in situ. Note normal mediastinal and cardiac silhouettes. ECG and pacing wires are also visible, surgical chest drains in situ

Approach via median sternotomy revealed the large aneurysm sac which was translucent to blood. The space occupying effect caused lateral deviation of the aorta, as well as inferior displacement of the heart into the diaphragm (Fig. 3). It also extended well into the left hemi-thorax, severely compromising the left lung. Cardiopulmonary bypass was established via aortic and bi-caval cannulation and the patient cooled to 34 °C. Dissection of the pulmonary artery allowed identification of the ligamentum arteriosus and safe dissection of the left recurrent laryngeal nerve (Fig. 4). Following dissection and preservation of the phrenic nerves the recipient heart and lungs were explanted. The donor heart-lung block was prepared and implanted and after haemostasis was confirmed, primary chest closure was performed. The patient was started on an induction immunosuppressive regime and transferred to the cardiac intensive care unit in a stable condition. A post-operative chest x-ray was performed which was normal (Fig. 5). The only significant post-operative complication was a below-knee deep vein thrombosis which was easily managed with enoxaparin. The patient was discharged 34d post-operatively and follow-up for 13 months since discharge has confirmed good progress.

## Discussion and conclusion

This case highlights the difficulties in managing patients with IPAH that are complicated by PAA. Timing of intervention for PAA is difficult as there is no evidence based guidelines on when this is best undertaken. For patients with IPAH complicated by PAA there is even less evidence to help guide decision making, and this case was complicated further by the extreme size of the aneurysm and the degree of right ventricular failure. Early double lung transplantation had been considered several years before the eventual heart-lung transplant, however the patient was clinically stable with medical management and did not qualify for listing at that time.

Bilateral lung transplant can be done in most pulmonary hypertension patients, even those with severe right ventricular dysfunction. Whilst bilateral lung transplantation has been utilised in the presence of PAA [18], the reason for considering heart and lung transplant in this scenario was due to the giant PAA size, severe pulmonary regurgitation due to annular dilatation and 4 cm pericardial effusion. The limitation of this approach is the increasing demand and short supply of suitable donor organs. The giant size of the aneurysm later in the disease progression meant lung transplantation would not have been possible in this case, and this would also not address the concurrent pulmonary valve dilatation and subsequent right ventricular failure. Another theoretical option would have been a graft repair of the aneurysm and pulmonary valve repair/replacement. However, this would have been extremely high risk, even more so in the presence of IPAH, and not feasible due to the extreme size of the aneurysm. Also, this would not address the patient’s progressive lung disease and would have potentially left the patient later requiring lung transplantation, a second major surgical intervention. Other examples of graft repair have been described for giant PAA [17] however, the aneurysm described in this patient is significantly larger than others reported, and none report such severe pulmonary valve disease and right heart failure. This case also highlights the importance of regular imaging to monitor the progression of IPAH complicated by PAA.

In conclusion, this case demonstrates that heart-lung transplantation is a good surgical option for difficult cases of IPAH complicated by giant PAA and right ventricular failure. However, this remains a procedure that is very rarely performed. The constant demand for organs and short supply of suitable donors means that there is a risk that these patients may not find a suitable donor before deterioration makes them unsuitable for transplantation. The use of extra corporeal membrane oxygenation has been successful as a bridge to transplant in cases of severe deterioration of IPAH [19]. Despite this, timing of intervention to optimise survival will continue to be a challenge in this complex group of patients.

## Data Availability

Not applicable.

## Abbreviations

PAH: Pulmonary arterial hypertension
IPAH: Idiopathic pulmonary arterial hypertension
PAA: Pulmonary artery aneurysm
